# AI-assisted accelerated MRI of the ankle: clinical practice assessment

**DOI:** 10.1186/s41747-023-00374-5

**Published:** 2023-10-20

**Authors:** Qiang Zhao, Jiajia Xu, Yu Xin Yang, Dan Yu, Yuqing Zhao, Qizheng Wang, Huishu Yuan

**Affiliations:** 1 Department of Radiology, Peking University Third Hospital, 49 North Garden Road, Haidian District, Beijing, 100191 People’s Republic of China; 2 United Imaging Research Institute of Intelligent Imaging, Beijing, People’s Republic of China

**Keywords:** Acceleration, Ankle, Artificial intelligence, Magnetic resonance imaging, Musculoskeletal diseases

## Abstract

**Background:**

High-spatial resolution magnetic resonance imaging (MRI) is essential for imaging ankle joints. However, the clinical application of fast spin-echo sequences remains limited by their lengthy acquisition time. Artificial intelligence-assisted compressed sensing (ACS) technology has been recently introduced as an integrative acceleration solution. We compared ACS-accelerated 3-T ankle MRI to conventional methods of compressed sensing (CS) and parallel imaging (PI) .

**Methods:**

We prospectively included 2 healthy volunteers and 105 patients with ankle pain. ACS acceleration factors for ankle protocol of T1-, T2-, and proton density (PD)-weighted sequences were optimized in a pilot study on healthy volunteers (acceleration factor 3.2–3.3×). Images of patients acquired using ACS and conventional acceleration methods were compared in terms of acquisition times, signal-to-noise ratio (SNR), contrast-to-noise ratio (CNR), subjective image quality, and diagnostic agreement. Shapiro-Wilk test, Cohen κ, intraclass correlation coefficient, and one-way ANOVA with post hoc tests (Tukey or Dunn) were used.

**Results:**

ACS acceleration reduced the acquisition times of T1-, T2-, and PD-weighted sequences by 32−43%, compared with conventional CS and PI, while maintaining image quality (mostly higher SNR with *p* < 0.004 and higher CNR with *p* < 0.047). The diagnostic agreement between ACS and conventional sequences was rated excellent (*κ* = 1.00).

**Conclusions:**

The optimum ACS acceleration factors for ankle MRI were found to be 3.2–3.3× protocol. The ACS allows faster imaging, yielding similar image quality and diagnostic performance.

**Relevance statement:**

AI-assisted compressed sensing significantly accelerates ankle MRI times while preserving image quality and diagnostic precision, potentially expediting patient diagnoses and improving clinical workflows.

**Key points:**

• AI-assisted compressed sensing (ACS) significantly reduced scan duration for ankle MRI.

• Similar image quality achieved by ACS compared to conventional acceleration methods.

• A high agreement by three acceleration methods in the diagnosis of ankle lesions was observed.

**Graphical Abstract:**

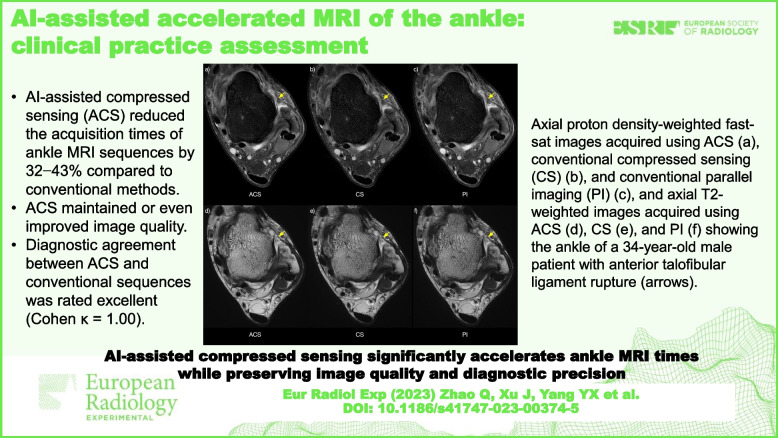

**Supplementary Information:**

The online version contains supplementary material available at 10.1186/s41747-023-00374-5.

## Background

Ankle injuries are among the most common musculoskeletal injuries in both the general public and physically active individuals engaged in various sports [1, 2]. Additionally, acute ankle sprains have a high recurrence rate, and up to 70% of patients may experience persistent physical impairment, including chronic ankle instability [3]. Ankle pathology can manifest acutely or chronically and might vary in tissue type, damage mode, and presentation. Magnetic resonance imaging (MRI) is generally accepted as a noninvasive approach for ankle imaging evaluation due to its reliability, safety, and other benefits over diagnostic arthroscopy. To visualize the complex anatomical structures within the ankle joints and to differentiate between various injury types, high in-plane resolution two-dimensional (2D) fast spin-echo (FSE) sequences need to be acquired along all the three dimensions [4]. However, the clinical application of high-resolution FSE sequences is currently limited by their lengthy acquisition periods and the motion artifacts associated [5]. By reducing MRI acquisition time, the efficiency of MRI exams can be increased, and patient comfort and compliance can be improved.

Various acceleration techniques have been developed to address this issue, such as parallel imaging (PI), partial Fourier imaging, and compressed sensing (CS). PI is commonly utilized in clinical applications, although at high acceleration factors, noise amplification can decrease the image quality [6]. Partial Fourier acceleration factor is typically limited to less than 2 fold, thus usually used in conjunction with other acceleration techniques [7]. CS, which leverages the sparsity constraint, offers a novel method for iterative reconstruction of under-sampled k-space data in a pseudorandom manner [8]. However, insufficient sparsity can result in aliasing artifacts that resemble noise, especially with an excessively high acceleration factor [9]. Efforts have been made to explore the application of artificial intelligence (AI), especially deep neural networks, in the reconstruction of undersampled k-space data, accelerating the MRI data acquisition without sacrificing image quality [10]. Recent research has demonstrated promising outcomes to create high-quality images in shorter time using a compressed sensing AI framework [11].

This study utilizes an AI-assisted compressed sensing (ACS) technology, which is recently presented to give an integrative MRI acceleration solution to address the limitations of the aforementioned techniques [9]. ACS employs an extended convolutional neural network (CNN) to collaborate with PI, partial Fourier, and CS, facilitating noise suppression, artifact reduction, and information recovery [12, 13]. The fully sampled k-space data are randomly undersampled and converted to image space as the model's input during the training phase. The model processes undersampled data to predict fully sampled data during the test phase.

This technique has been previously reported as helpful in abdominal imaging of the liver and kidney [9, 14]. Nevertheless, the clinical application of ACS has remained limited in ankle imaging, even though in this discipline, high-resolution 2D imaging is required to visualize even subtle pathologies of joint structures. Therefore, the objective of the current study was to assess the effectiveness of using ACS to accelerate ankle imaging. Conventional 2D sequences for the ankle were acquired using PI, CS, and ACS to determine whether ACS could reduce the overall scan time while maintaining diagnostic image quality.

## Methods

### Study population

The study was conducted between June and October 2022. One hundred five patients (55 men and 50 women; age, 37.9 ± 11.4 years) with a complaint of ankle pain who had undergone ankle MRI were included (left ankle, 43; right ankle, 62). The institutional review board approved this prospective study. Written informed consent was obtained from all study participants, following local ethical regulations. Exclusion criteria for study participation were age < 18 years, pregnancy, and ankle surgery within 6 months.

### ACS image reconstruction

The ACS method is a United States Food and Drug Administration-approved method for accelerating MRI acquisition using a deep learning approach, *i.e.*, convolutional neural networks (CNN). While CNN-based methods have shown superior reconstruction quality, their performance and reliability in clinical settings are often uncertain due to the black-box nature of the network. To address this uncertainty, ACS integrates the output of the trained AI module as an additional constraint into the CS framework. This is achieved by adding a regularization term based on the difference between the reconstructed image and the predicted image of the AI module, as indicated in Eq. 1:

1 $${\text{argmin}}_{x}{\text{||}Ex-y||}_{2}^{2}+{\lambda }_{1}{|\left|\psi \left(x\right)\right||}_{1}+{\lambda }_{2}{|\left|\phi \left({x}_{AI},x\right)\right||}_{w}+{\lambda }_{3}{|\left|PI\right||}_{m}+{\lambda }_{4}{|\left|PF\right||}_{n}$$

where $x$ denotes the image to be reconstructed, $E$ denotes the production of Fourier encoding with binary k-space sampling mask, $y$ represents the acquired $k$ space data, $\psi$ denotes the sparse thansform, ${x}_{AI}$ is the reconstructed image of the trained AI module, and ${\lambda }_{i}$ is the constrain for each term.

The ACS neural networks were trained using a dataset of two million fully sampled images, which were previously acquired from both phantoms (2%) and human volunteers (98%) [13]. Meanwhile, the architectural design employed in the iteration processes was derived from the k-space, with multiscale sparsification integrated. The CS, partial Fourier, and PI are all incorporated in the mathematical model. Simulation tests [13] have demonstrated that ACS is able to correct errors in the output generated by the AI model and achieve high consistency compared to the fully-sampled reference standard.

The architecture of the deep neural network used for image reconstruction in this study was an extended fully CNN with paired undersampled and full-sampled images. After applying the inversed Fourier transform to the k-space signal, the network took the real and imaginary components of the undersampled images as the input and produced the real and imaginary components of the reconstructed images, respectively. The network used in this study resembled U-net [15], with the modifications that residual blocks [16], which consisted of two convolution operations and a skipping connection, substituted the convolution operation in the original U-net. To speed up learning progress, a long skipping connection was also incorporated to learn the residual between the fully sampled and under-sampled images. To further enhance the quality of the reconstructed images, the least squared generative adversarial network (GAN) training technique was employed [17].

### Imaging protocol and study design

All subjects were examined with a clinical 3.0-T scanner (uMR 880, United Imaging Healthcare, Shanghai, China) with a dedicated 24-channel receive ankle coil. The protocol included a T2-weighted FSE sequence, a T1-weighted FSE sequence, and three proton density (PD)-weighted FSE sequences with fat saturation (fat-sat) acquired with acceleration techniques of PI, CS, and ACS as listed in Table 1.

**Table 1:** Parameters of the sequences acquired using PI, CS, and ACS

**Sequence**	**PD-weighted FSE fat-sat**			**T2-weighted FSE**			**PD-weighted FSE fat-sat**			**T1-weighted FSE**			**PD-weighted FSE fat-sat**		
**Plane**	**Transversal**			**Transversal**			**Coronal**			**Coronal**			**Sagittal**		
Acceleration	PI	CS	ACS	PI	CS	ACS	PI	CS	ACS	PI	CS	ACS	PI	CS	ACS
Field of view (mm)	150	150	150	150	150	150	150	150	150	150	150	150	150	150	150
Slice thickness (mm)	2	2	2	2	2	2	2	2	2	2	2	2	3	3	3
Acquisition matrix	384 × 384	384 × 384	384 × 384	384 × 384	384 × 384	384 × 384	336 × 336	336 × 336	336 × 336	320 × 400	320 × 400	320 × 400	302 × 336	302 × 336	302 × 336
Repetition time (ms)	3,861	3,861	3,861	7,800	7,800	7,800	4,406	4,406	4,406	765	765	765	3,115	3,115	3,115
Echo time (ms)	32.16	32.16	32.96	79.56	79.56	77.2	46.08	46.08	46.96	7.92	7.92	7.52	44.4	44.4	45.2
Refocusing flip angle (°)	120	120	120	120	120	120	90	90	90	120	120	120	135	135	135
Bandwidth (Hz/Pixel)	260	260	260	300	300	300	150	150	150	320	320	320	150	150	150
Echo train length	7	7	7	20	20	20	7	7	7	3	3	3	7	7	7
Acceleration factor	2	2.1	3.3	2	2.1	3.3	2	2.1	3.2	2	2.1	3.2	2	2.1	3.3
Acquisition time (min:s)	2:10	1:56	1:14	1:36	1:29	0:55	1:38	1:24	0:56	1:36	1:24	0:57	2:21	2:09	1:21

*ACS* Artificial intelligence-assisted compressed sensing, *CS* Compressed sensing, *FSE* Fast spin-echo, *PD* Proton density, *PI* Parallel imaging

This study consisted of two steps: (1) a pilot study on healthy volunteers to explore the optimal acceleration factors for ACS and (2) an assessment of a cohort of patients with ankle injuries. The images acquired with PI (acceleration factor of 2.0×) and CS (acceleration factor of 2.1×) were used as baseline. The acceleration factor of ACS was varied from 2.3× to 3.8×.

### Pilot study on healthy volunteers

Two healthy volunteers were scanned to confirm the acceleration factors for ACS. The main focus was the maximum reduction of scan time without introducing artifacts or altering anatomical morphology. A series of ACS scans with acceleration factors ranging from 2.3× to 3.8× were acquired. To compare the ACS images to the PI (2.0×) and CS (2.1×) images for each sequence and provide a subjective evaluation, two independent radiologists, with 5 (Q.W.) and 8 years (Y.Z.) of experience in musculoskeletal radiology, rated the image quality with a standardized 5-point Likert-scale scoring system for a thorough assessment based on their preferences [18].

### Assessment of a cohort of patients with ankle injuries

#### Quantitative image analysis

For anatomical structural quantitative image comparison, regions of interest (ROIs) in identical locations on images acquired with PI, CS, and ACS were delineated by the two independent abovementioned experienced radiologists (Q.W. and Y.Z.). The ROIs were drawn on the subchondral bone, joint fluid, cartilage, ligaments, muscle, fat, and tendons. Due to the ankle ligament alignment and the morphological structure of the ligaments, only the axial sequences were used to delineate ligament ROIs. The signal-to-noise ration (SNR) and contrast-to-noise ratio (CNR) measurements were calculated from the ROIs. SNR was calculated by dividing the average signal intensity (SI) value of ROI placed on tissue (*SI*_*tissue*_) by the standard deviation (SD) of SI of the tissue ROI (*SD*_*tissue*_). SD of the tissue SI was used instead of background SI as SNR calculated by the background SD is not uniform across the regions of an accelerated sparse image [19, 20]. After acquiring the SNR for these structures, CNR was calculated for cartilage/fluid, cartilage/subchondral bone, ligament/fluid, ligament/fat, tendon/fluid, and tendon/muscle using the following Eq. [19]:

2 $$CNR=\frac{{SI}_{{tissue}_{1}}-{SI}_{{tissue}_{2}}}{\sqrt{{SD}_{{tissue}_{1}}^{2}+{SD}_{{tissue}_{2}}^{2}}}$$

#### Qualitative image analysis

The image quality of the PI, CS, and ACS images was evaluated in a blinded manner by the above-mentioned musculoskeletal radiologists. All images were randomized and displayed simultaneously as images A, B, and C using a commercially available picture archiving and communication system workstation. Optimal adjustments were made to the window widths, contrasts, and levels for each sequence. The subjective image quality was rated using a five-point Likert scale regarding the depiction of anatomic structures (5 = excellent, optimal diagnostic value and clearly shows the structure with nearly no artifacts; 4 = good, good for the majority of diagnoses, with structures shown with minor artifacts; 3 = fair, acceptable for the majority of diagnoses with the evaluation of the structure somewhat limited; 2 = limited, with severe localized artifacts and noise and the assessment of the structure substantially limited; 1 = poor, with extensive artifacts and noise, barely able to show structures).

#### Diagnostic agreement analysis

The two radiologists assessed images acquired with PI, CS, and ACS accelerations in a randomized order. The ligament injuries were evaluated depending on the degree of tearing of the anterior talofibular ligament and calcaneofibular ligament separately, depending on the presence of high signal within the ligament, abnormal shape or orientation of the ligament, and discontinuous signal of the ligament, with a three-point scale: 0 = no lesion; 1 = partial-thickness tear; and 2 = complete tear. Osteochondral lesions were also assessed with a five-point scale [21]: 0 = no lesion; 1 = hyperintense but morphologically intact cartilage surface; 2 = fibrillation or fissures not extending to the bone; 3 = flap present or bone exposed; 4 = loose undisplaced fragment; and 5 = displaced fragment.

### Statistical analysis

Descriptive statistics presented are mean ± standard error of mean (SEM) for continuous variables (means ± standard deviatons are given in Supplementary Tables), and median (25th–75th percentile) for discrete variable, and the normality was assessed using the Shapiro-Wilk test. Inter-reader agreements for image quality were measured by Cohen κ statistics, while interobserver agreements for the SNR and CNR were examined by calculating the intraclass correlation coefficient (ICC). SNRs, CNRs, and subjective image quality ratings between pairs of sequences acquired using PI, CS, and ACS were quantitatively assessed using the one-way ANOVA with Tukey’s post hoc tests or Friedman with Dunn’s post hoc tests, as appropriate. The evaluation of various ankle joint pathologies was compared between sequences acquired using PI, CS, and ACS. The agreement of diagnostic performance was evaluated with Fleiss’ κ statistic. All statistical analyses were performed using SPSS version 26, released 2019 (IBM, Armonk, NY, USA); *p* values lower than 0.05 were considered statistically significant.

## Results

### Patients’ characteristics

Two healthy volunteers and 105 patients were included in this study, with 103 ligament injuries and 34 osteochondral lesions.

### Pilot study on healthy volunteers

**Fig. 1:**
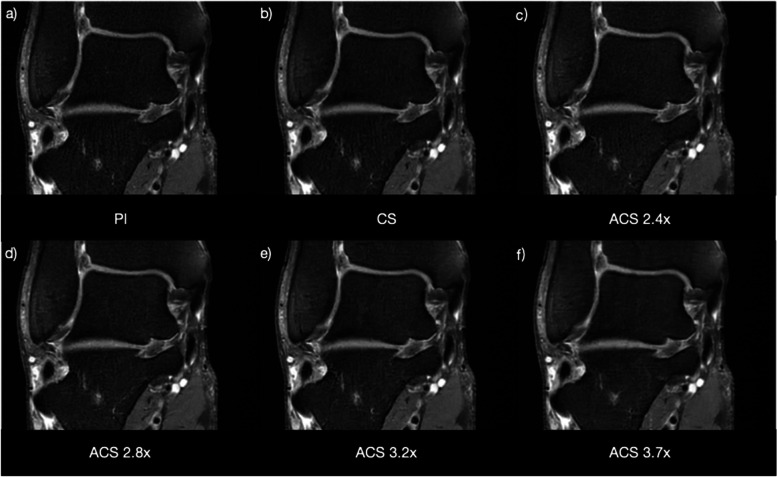
Coronal PD fat-sat images on a healthy volunteer acquired using PI (**a**), CS (**b**), ACS 2.4× (**c**), ACS 2.8× (**d**), ACS 3.2× (**e**), and ACS 3.7× (**e**). *ACS* Artificial intelligence-assisted compressed sensing, *CS* Compressed sensing, *PD* Proton density, *PI* Parallel imaging

Though the tendons and the tibial nerve were clearly depicted on all images, significant blurring artifacts were observed as the acceleration factors increased to 3.7, as illustrated in Fig. 1. Therefore, both radiologists favored the ACS acceleration factors of 3.2–3.3× for PD-weighted FSE, T1-weighted FSE, and T2-weighted FSE sequences (Table 1).

### Baseline characteristics

The total acquisition time was 5:23 min:s for ankle protocol with ACS. Compared to conventional 2D sequences accelerated using PI, the ACS technique reduced the acquisition time to 57% of that with PD-FSE sequence (acquisition time 6:09 min:s), 59% of that with T1-weighted FSE sequence (acquisition time 1:36 min:s), and 57% of that with T2-weighted FSE sequence (acquisition time 1:36 min:s). Compared to CS acceleration, the ACS technique reduced the acquisition time to 62–67% of that with PD-weighted FSE sequence (acquisition time 5:29 min:s), to 67% of that with T1-weighted FSE sequence (acquisition time 1:24 min:s), and to 62% of that with T2-weighted FSE sequence (acquisition time 1:29 min:s).

### Quantitative image analysis

**Fig. 2:**
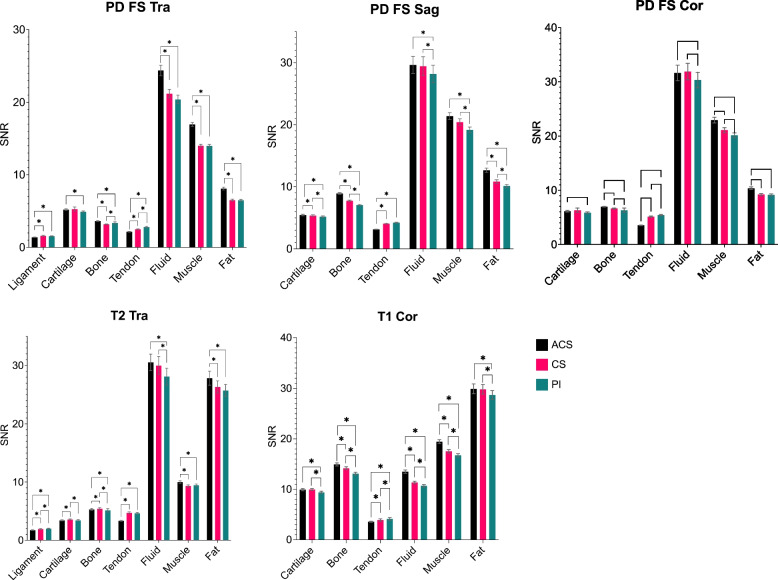
SNR measurements of different anatomical structures derived from sequences with accelerations of ACS, CS, and PI. Statistically different pairs (*p* < 0.05) are marked with the star signs. *ACS* Artificial intelligence-assisted compressed sensing, *CS* Compressed sensing, *PI* Parallel imaging, *SNR* Signal-to-noise ratio

The statistical results for the SNRs in the selected tissues of ACS, PI, and CS sequences are shown in Fig. 2 and Table 2, with the statistical analysis result in Table S2. Images acquired with ACS had significantly higher SNRs (*p* ≤ 0.004) than those with PI in cartilage (for all sequences except for transversal T2-weighted), subchondral bone (for all sequences), fluid (for all sequences), muscle (for all sequences), and fat (for all sequences), but with significant lower SNRs (*p* ≤ 0.001) in ligament (for transversal PD-weighted fat-sat and transversal T2-weighted) and tendon (for all sequences). Images acquired with ACS had significantly higher SNRs than those with CS in the subchondral bone (for all sequences), fluid (for transversal PD-weighted fat-sat and coronal T1-weighted), muscle (for all sequences except for sagittal PD-weighted fat-sat), and fat (for all sequences except for coronal T1-weighted). However, the SNRs of tendons were found to be lower when compared with those with CS accelerations for all sequences (*p* ≤ 0.005).

**Table 2:** Signal-to-noise ratio of ligament, cartilage, subchondral bone, tendon, fluid, muscle, and fat

**PD-weighted fat-sat transversal**							
	Ligament	Cartilage	Subchondral bone	Tendon	Fluid	Muscle	Fat
ACS	1.40 ± 0.04	5.22 ± 0.11	3.64 ± 0.06	2.16 ± 0.05	24.39 ± 0.71	16.95 ± 0.28	8.09 ± 0.19
CS	1.60 ± 0.09	5.28 ± 0.27	3.19 ± 0.04	2.49 ± 0.06	21.20 ± 0.57	14.01 ± 0.20	6.49 ± 0.13
PI	1.56 ± 0.04	4.93 ± 0.09	3.38 ± 0.14	2.81 ± 0.08	20.41 ± 0.58	13.99 ± 0.20	6.47 ± 0.13
**PD-weighted fat-sat sagittal**							
	Cartilage	Subchondral bone	Tendon	Fluid	Muscle	Fat	
ACS	5.46 ± 0.12	8.90 ± 0.13	3.14 ± 0.04	29.63 ± 1.39	21.38 ± 0.55	12.65 ± 0.35	
CS	5.37 ± 0.12	7.72 ± 0.10	4.06 ± 0.07	29.43 ± 1.51	20.42 ± 0.53	10.82 ± 0.29	
PI	5.19 ± 0.11	7.05 ± 0.09	4.22 ± 0.07	28.17 ± 1.4	19.16 ± 0.45	10.11 ± 0.23	
**PD-weighted fat-sat coronal**							
	Cartilage	Subchondral bone	Tendon	Fluid	Muscle	Fat	
ACS	6.13 ± 0.14	6.96 ± 0.07	3.55 ± 0.05	31.62 ± 1.43	22.92 ± 0.51	10.38 ± 0.21	
CS	6.33 ± 0.39	6.62 ± 0.08	5.13 ± 0.09	31.88 ± 1.50	21.07 ± 0.47	9.21 ± 0.19	
PI	5.87 ± 0.14	6.68 ± 0.07	5.41 ± 0.11	30.33 ± 1.43	20.15 ± 0.41	9.15 ± 0.17	
**T2-weighted transversal**							
	Ligament	Cartilage	Subchondral bone	Tendon	Fluid	Muscle	Fat
ACS	1.74 ± 0.05	3.41 ± 0.10	5.30 ± 0.17	3.35 ± 0.08	30.53 ± 1.41	9.98 ± 0.24	27.84 ± 1.18
CS	1.92 ± 0.08	3.56 ± 0.10	5.41 ± 0.18	4.72 ± 0.16	29.98 ± 1.53	9.32 ± 0.19	26.34 ± 1.05
PI	1.99 ± 0.06	3.43 ± 0.10	5.14 ± 0.28	4.62 ± 0.15	28.1 ± 1.42	9.46 ± 0.20	25.75 ± 1.03
**T1-weighted coronal**							
	Cartilage	Subchondral bone	Tendon	Fluid	Muscle	Fat	
ACS	9.94 ± 0.21	14.94 ± 0.31	3.57 ± 0.10	13.51 ± 0.33	19.46 ± 0.37	29.93 ± 0.96	
CS	9.98 ± 0.18	14.2 ± 0.29	3.88 ± 0.26	11.35 ± 0.24	17.54 ± 0.33	29.82 ± 0.95	
PI	9.41 ± 0.20	13.12 ± 0.26	4.13 ± 0.26	10.71 ± 0.24	16.75 ± 0.32	28.70 ± 0.88	

Data given as means ± standard error of means. *ACS* Artificial intelligence-assisted compressed sensing, *CS* Compressed sensing, *PD* Proton density, *PI* Parallel imaging

**Fig. 3:**
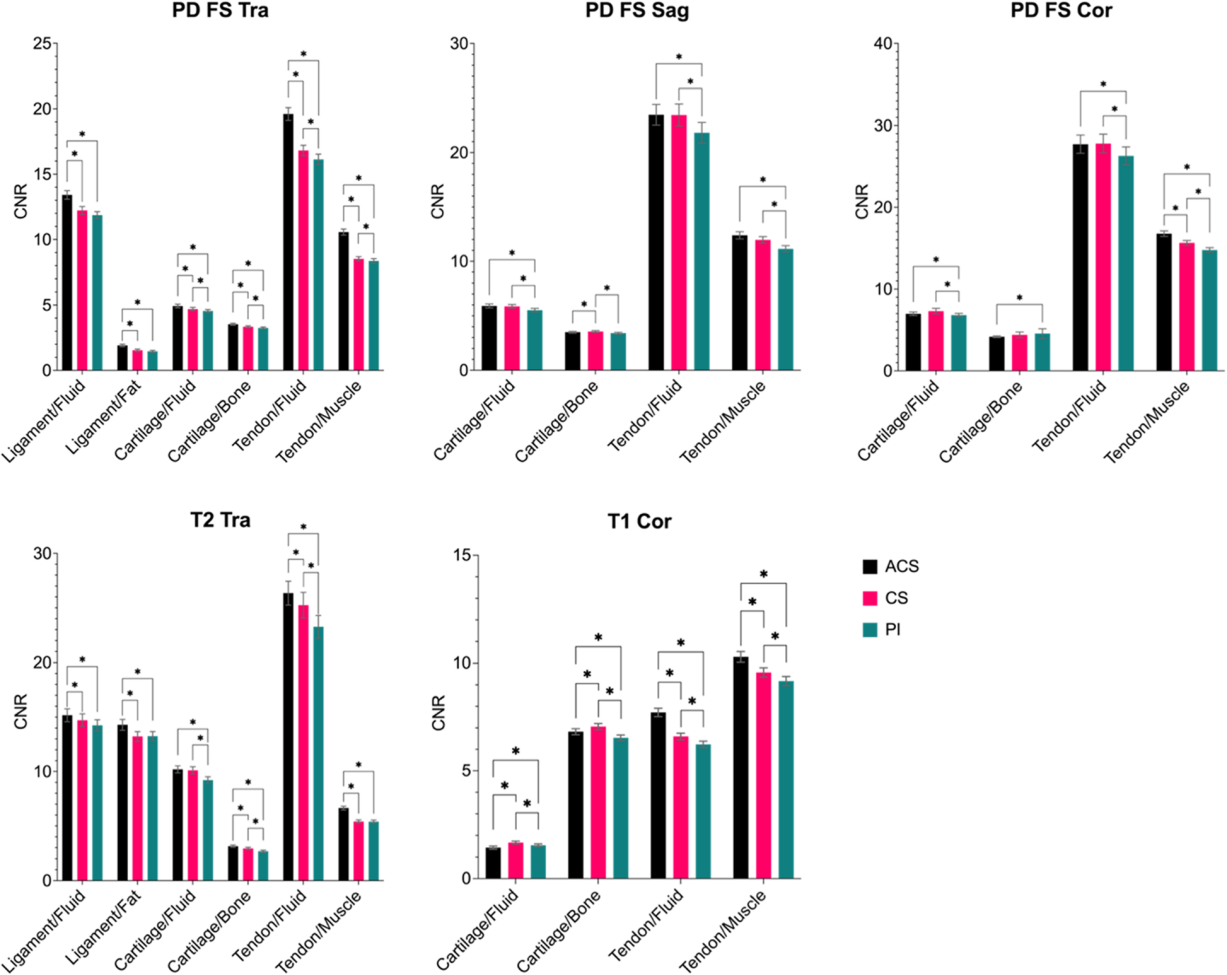
CNR measurements of different anatomical structure pairs derived from sequences with accelerations of ACS, CS, and PI. Statistically different pairs (*p* < 0.05) are marked with the star signs. *ACS* Artificial intelligence-assisted compressed sensing, *CS* Compressed sensing, *PI* Parallel imaging, *SNR* Signal-to-noise ratio

The CNRs (Fig. 3 and Table 3, with the statistical analysis result in Table S4.) of ligament against both fluid and fat calculated from ACS sequences were significantly higher than those with PI (*p* ≤ 0.047). The CNRs of cartilage/fluid were higher (*p* ≤ 0.001) on transversal and sagittal PD-weighted fat-sat and transversal T2-weighted sequences but lower on coronal T1-weighted and coronal PD-weighted fat-sat for ACS compared to conventional PI (*p* ≤ 0.017). Significant higher CNRs (*p* ≤ 0.001) were found for ACS compared with PI in cartilage/subchondral bone (all sequences except for sagittal and coronal PD-weighted fat-sat), tendon/fluid (for all sequences), and tendon/muscle (for all sequences). Compared with CS acceleration, significant higher CNRs (*p* ≤ 0.001) were calculated for ACS images in ligament/fluid (transversal PD-weighted fat-sat and transversal T2-weighted), ligament/fat (transversal PD-weighted fat-sat and transversal T2-weighted), cartilage/fluid (transversal PD-weighted fat-sat), cartilage/subchondral bone (for all sequences except for coronal PD-weighted fat-sat and coronal T1-weighted), tendon/fluid (transversal PD-weightedfat-sat, transversal T2-weighted and coronal T1-weighted), and tendon/muscle (for all sequences except for sagittal PD-weighted fat-sat). However, on coronal T1-weighted images, lower CNRs were found for cartilage against fluid and subchondral bone (*p* ≤ 0.001).

**Table 3:** Contrast-to-noise ratio of ligament/fluid, ligament/fat, cartilage/fluid, cartilage/subchondral bone, tendon/fluid, and tendon/muscle

**PD-weighted fat-sat transversal**						
	Cartilage/fluid	Cartilage/subchondral bone	Tendon/fluid	Tendon/muscle	Ligament/fluid	Ligament/fat
ACS	4.93 ± 0.13	3.53 ± 0.07	19.60 ± 0.48	10.56 ± 0.23	13.41 ± 0.32	1.91 ± 0.09
CS	4.69 ± 0.13	3.34 ± 0.06	16.81 ± 0.40	8.51 ± 0.17	12.21 ± 0.30	1.53 ± 0.09
PI	4.54 ± 0.12	3.25 ± 0.06	16.13 ± 0.39	8.35 ± 0.17	11.86 ± 0.26	1.46 ± 0.07
**PD-weighted fat-sat sagittal**						
	Cartilage/fluid	Cartilage/subchondral bone	Tendon/fluid	Tendon/muscle		
ACS	5.91 ± 0.18	3.49 ± 0.08	23.47 ± 0.94	12.38 ± 0.32		
CS	5.87 ± 0.17	3.56 ± 0.08	23.45 ± 1.00	11.96 ± 0.29		
PI	5.52 ± 0.16	3.40 ± 0.08	21.82 ± 0.95	11.14 ± 0.29		
**PD-weighted fat-sat coronal**						
	Cartilage/fluid	Cartilage/subchondral bone	Tendon/fluid	Tendon/muscle		
ACS	6.98 ± 0.21	4.16 ± 0.10	27.71 ± 1.11	16.75 ± 0.33		
CS	7.30 ± 0.34	4.40 ± 0.35	27.79 ± 1.15	15.62 ± 0.30		
PI	6.81 ± 0.21	4.55 ± 0.60	26.28 ± 1.10	14.75 ± 0.28		
**T2-weighted transversal**						
	Cartilage/fluid	Cartilage/subchondral bone	Tendon/fluid	Tendon/muscle	Ligament/fluid	Ligament/fat
ACS	10.19 ± 0.32	3.13 ± 0.10	26.35 ± 1.09	6.64 ± 0.17	16.51 ± 0.72	15.19 ± 0.54
CS	10.10 ± 0.33	2.92 ± 0.10	25.25 ± 1.17	5.40 ± 0.15	15.84 ± 0.73	14.01 ± 0.54
PI	9.20 ± 0.30	2.66 ± 0.09	23.26 ± 1.03	5.39 ± 0.15	14.87 ± 0.61	14.03 ± 0.53
**T1-weighted coronal**						
	Cartilage/fluid	Cartilage/subchondral bone	Tendon/fluid	Tendon/muscle		
ACS	1.42 ± 0.07	6.82 ± 0.13	7.71 ± 0.19	10.29 ± 0.24		
CS	1.65 ± 0.07	7.06 ± 0.13	6.60 ± 0.15	9.57 ± 0.21		
PI	1.53 ± 0.06	6.53 ± 0.13	6.23 ± 0.14	9.17 ± 0.21		

Data given as means ± standard error of means. *ACS* Artificial intelligence-assisted compressed sensing, *CS* Compressed sensing, *PD* Proton density, *PI* Parallel imaging

### Qualitative image analysis

**Fig. 4:**
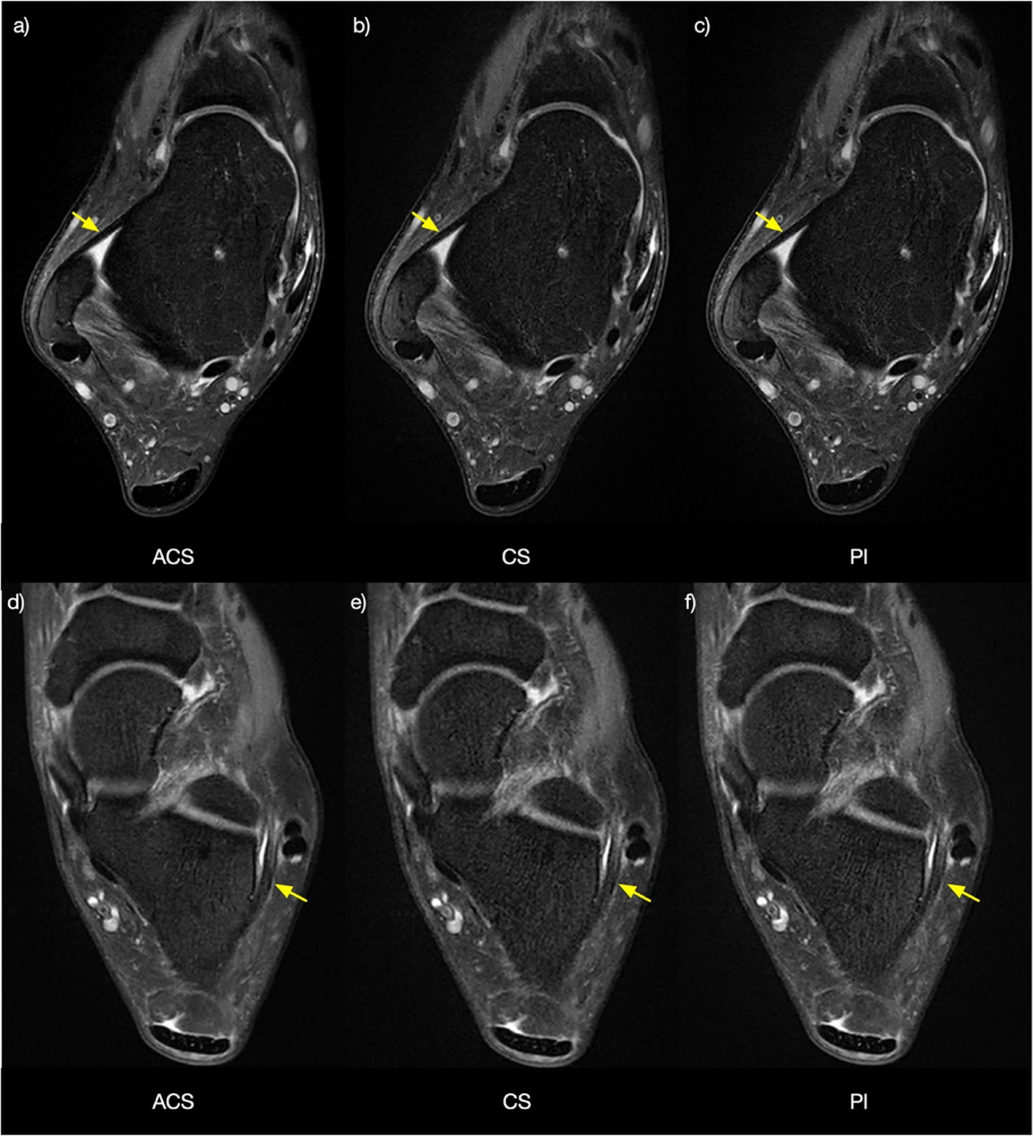
Axial PD-weighted fat-sat images acquired using ACS (**a**, **d**), CS (**b**, **e**), and PI (**c**, **f**). The quality of images showing the anterior talofibular ligament (arrows in **a**, **b**, and **c**) and the calcaneofibular ligament (arrows in **d**, **e**, and **f**) was rated equally (score 5) on all the images by both readers. *ACS* Artificial intelligence-assisted compressed sensing, *CS* Compressed sensing, *PD* Proton density, *PI* Parallel imaging

Image quality ratings were performed for each sequence and all participants (Table 4). Similar image quality was noticed in general across three acceleration methods (Fig. 4). Images acquired with ACS showed significantly higher ratings for structures of the anterior and posterior talofibular ligaments and calcaneofibular ligament than CS and PI (*p* = 0.015 and *p* < 0.001, respectively). In addition, ACS yielded significantly higher image quality for anterior and posterior tibiofibular ligaments than PI (*p* = 0.009).

**Table 4:** Comparison of the quality of structures of the ankle of images acquired with ACS, CS, and PI

	Reader 1			Reader 2		
	ACS	CS	PI	ACS	CS	PI
**Ligament**						
Anterior talofibular	5 (5–5)	5 (5–5)	5 (5–5)	5 (5–5)	5 (4–5)	4 (4–5)
Posterior talofibular	5 (5–5)	5 (5–5)	5 (5–5)	5 (5–5)	5 (4–5)	4 (4–5)
Calcaneofibular	5 (5–5)	5 (4–5)	5 (4–5)	5 (5–5)	5 (4–5)	4 (4–5)
Deep medial collateral	5 (5–5)	5 (5–5)	5 (5–5)	5 (5–5)	5 (5–5)	5 (5–5)
Superficial medial collateral	5 (5–5)	5 (5–5)	5 (5–5)	5 (5–5)	5 (5–5)	5 (5–5)
Anterior tibiofibular	5 (5–5)	5 (5–5)	5 (5–5)	5 (5–5)	5 (5–5)	5 (5–5)
Posterior tibiofibular	5 (5–5)	5 (5–5)	5 (4–5)	5 (5–5)	5 (5–5)	5 (4–5)
**Cartilage**						
Tibiofibular	5 (5–5)	5 (5–5)	5 (5–5)	5 (5–5)	5 (5–5)	5 (5–5)
Talar	5 (5–5)	5 (5–5)	5 (5–5)	5 (5–5)	5 (5–5)	5 (5–5)
**Tendon**						
Extensor	5 (5–5)	5 (4–5)	5 (4–5)	5 (5–5)	5 (5–5)	5 (5–5)
Peroneal	5 (5–5)	5 (5–5)	5 (5–5)	5 (5–5)	5 (5–5)	5 (5–5)
Flexor	5 (5–5)	5 (5–5)	5 (5–5)	5 (5–5)	5 (5–5)	5 (5–5)
**Subchondral bone**						
Talus	5 (5–5)	5 (5–5)	5 (5–5)	5 (5–5)	5 (5–5)	5 (5–5)
Fibula	5 (5–5)	5 (5–5)	5 (5–5)	5 (5–5)	5 (5–5)	5 (5–5)
Tibia	5 (5–5)	5 (5–5)	5 (5–5)	5 (5–5)	5 (5–5)	5 (5–5)

5-point Likert scale (1 = worst; 5 = best). Data given as median (25th–75th percentile). *ACS* Artificial intelligence-assisted compressed sensing, *CS* Compressed sensing, *PI* Parallel imaging

### Interobserver agreement

The interobserver agreement between two readers in scoring image quality was substantial consistently for ACS accelerated sequences and routine sequences (κ = 0.637−0.875, *p* < 0.001). The ICC between the two readers was 0.989 for SNR and 0.987 for CNR (95% confidence interval 0.984–0.993 and 0.979–0.992, respectively).

### Diagnostic agreement

**Fig. 5:**
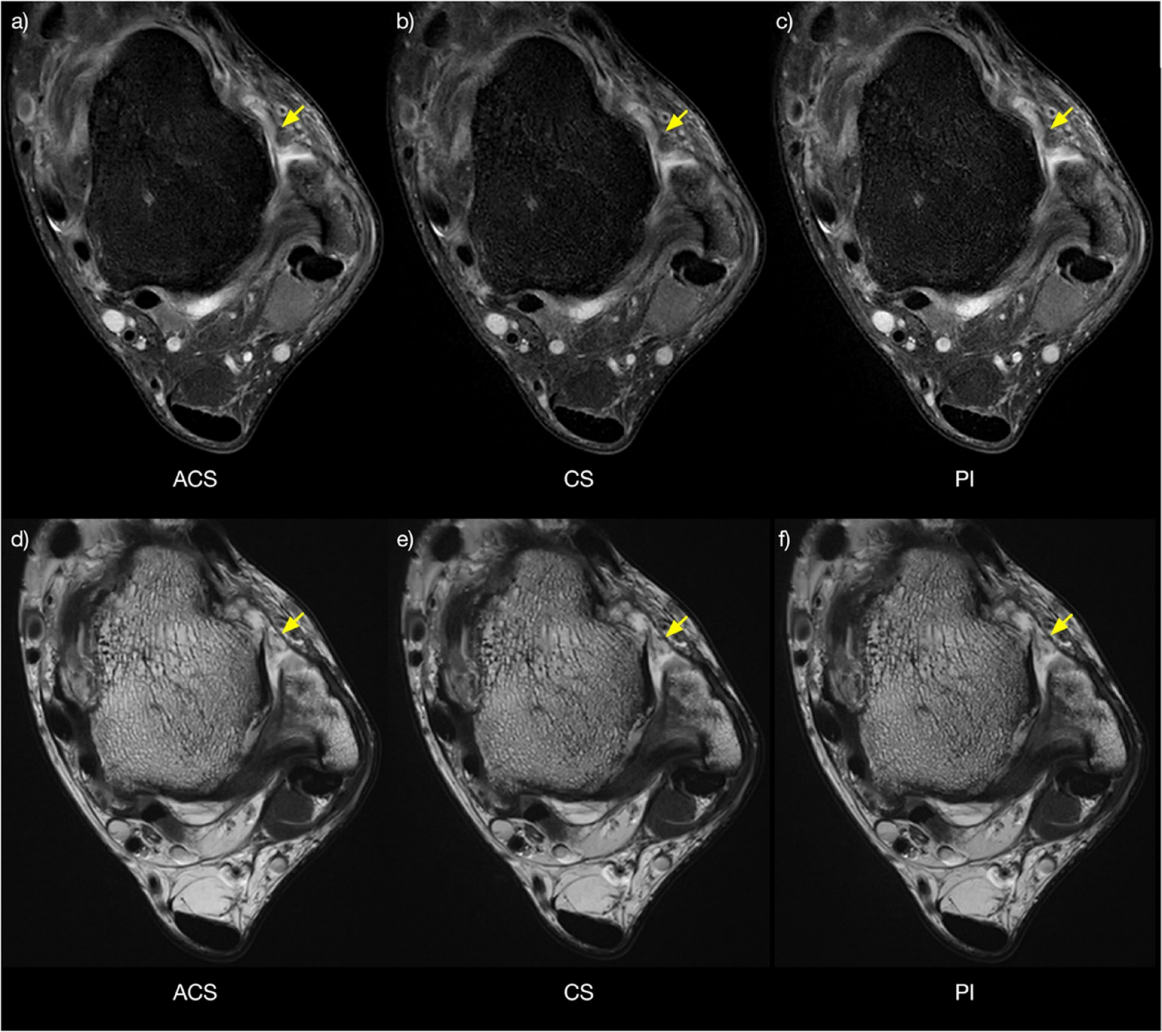
Axial PD-weighted fast-sat images acquired using ACS (**a**), CS (**b**), and PI (**c**) and axial T2-weighted images acquired using ACS (**d**), CS (**e**), and PI (**f**) showing the ankle of a 34-year-old male patient with anterior talofibular ligament rupture (arrows). *ACS* Artificial intelligence-assisted compressed sensing, *CS* Compressed sensing, *PD* Proton density, *PI* Parallel imaging

**Fig. 6:**
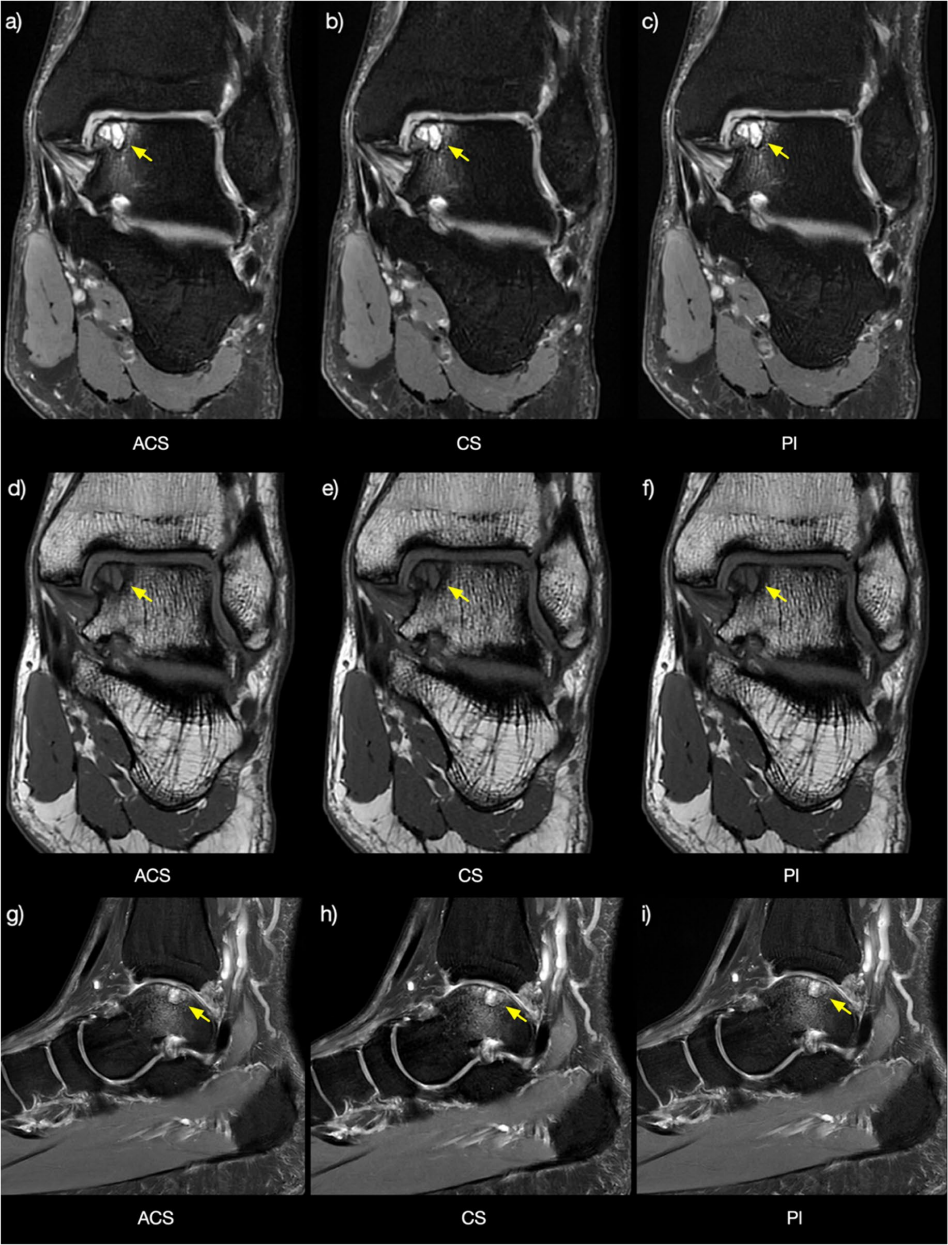
Coronal PD-weighted fat-sat and T1-weighted images acquired using ACS (**a**, **d**), CS (**b**, **e**), and PI (**c**, **f**) showing the ankle of a 34-year-old male patient with an osteochondral lesion (arrows, grade 4). Sagittal PD-weighted fat-sat images acquired with ACS (**g**), CS (**h**), and PI (**i**) showing bone marrow changes adjacent to the osteochondral lesion (arrows). *ACS* Artificial intelligence-assisted compressed sensing, *CS* Compressed sensing, *PD* Proton density, *PI* Parallel imaging

Of the 103 patients with ligament lesions and 34 patients with osteochondral lesions, 32 patients showed both ligament and osteochondral lesions (Figs. 5 and 6). The evaluation of ankle pathologies resulted in an agreement of *κ* = 1.00 across sequences acquired with ACS, CS, and PI. The interobserver reliability was excellent for all criteria (*κ* = 0.96–1.00).

## Discussion

Our study has demonstrated the capability of compressed sensing combined with parallel imaging, partial Fourier, and deep learning reconstruction (ACS) to reduce examination time without significantly compromising SNR, CNR, image quality, or diagnostic confidence level for ankle MRI compared to conventional PI and CS accelerations. This study evaluates the utility of the abovementioned AI-assisted acceleration technique for routine clinical ankle MRI.

Conventional PI acceleration uses the spatial sensitivity of each receiver in a multicoil array, reconstructs images from undersampled k-space data, and reduces acquisition time [6, 22]. CS provides a novel approach to recover the image information from undersampled k-space. Previous studies showed that musculoskeletal MRI with CS acceleration could reduce scan time while maintaining image quality for both 2D and three-dimensional sequences [23–26]. Our comparison between CS and PI also showed that CS is favored to reduce examination time in routine clinical practice. However, one drawbacks of CS is the challenge of finding appropriate sparsity for specific applications. The hyperparameters usually need to be tuned manually, which is both time-consuming and difficult to standardize [27, 28]. Recent developments in machine learning techniques that enable faster imaging address CS drawbacks. These methods operate in image space, incorporate measured coil sensitivities in the reconstruction, and generalize the concept of compressed sensing by learning the entire reconstruction procedure for MRI data [28–30]. Hammernik et al. [27] and Knoll et al. [30] proposed a “variational” network based on CNN, essentially a deep learning extension of PI and CS, and demonstrated successful reconstruction of accelerated knee images [28, 31]. Liu et al. [28] developed a GAN-based reconstruction model [32] to preserve the natural appearance of the images, where a limitation with pure CNN reconstruction lies in the over smoothness of the generated images [33]. The ACS technique used in our study incorporates both CNN and GAN structures to enhance the quality of the reconstructed images. Our results further demonstrate that the deep learning-based reconstruction method has superior potential to reduce MRI examination time compared to conventional PI and CS in routine clinical practice.

The ACS technique has been previously reported in the clinical applications for fast T2-weighted abdominal imaging of the liver and kidney. Much shorter scan times compared to conventional abdominal imaging sequences significantly reduced motion artifacts, hence providing better image quality and diagnostic confidence level [9, 14]. In this study, we showed that not only T2-weighted sequences but also T1- and PD-weighted sequences benefit from the novel deep learning-based acceleration technique. This study has also extended the clinical applications to musculoskeletal MRI, where high-resolution images are crucial for depicting different anatomical structures and pathologies.

In accordance with our hypothesis, the ACS sequences yielded almost the same image quality as the conventional PI and CS sequences. Moreover, slightly higher average subjective image quality ratings for ligaments were found in ACS images than in CS and PI images. The quantitative analysis also indicated that the ACS sequences could yield better SNR and CNR for most tissues than CS and PI sequences. However, the differences in SNR and CNR (< 3 arbitrary units) and image quality ratings (< 5%) were minor, resulting in a high agreement in the assessment of pathologies of the ankles between acceleration methods.

Unlike previous studies on the combined CS and PI acceleration for ankle imaging that mainly were performed on healthy volunteers [24, 25], this study assessed ACS acceleration with ankle-injured patients in actual clinical practice. In a related study [11] aimed at evaluating the efficacy of AI framework in accelerating ankle imaging, a comparable time reduction of the overall scan duration as our study was demonstrated, although diagnostic image quality was not consistently maintained. There is a large difference between the acceleration factor used in this study and our acceleration factor. However, the direct comparison of acceleration factors would be difficult considering several aspects such as different sequence designs, scanner hardware, and coils. Both acceleration methods employ CNN models addition to CS framework. The iterative reconstruction in ACS is for the whole CS framework with the trained AI module as an additional constraint, while in the architecture of the previous study, iterations are mimicked by deep learning network in ISTA-Net [34].

Common lesions within the anterior talofibular ligament, calcaneofibular ligament, and cartilage were evaluated separately in our study, leading to results that are more specific. Our results provide reliable and comprehensive evidence supporting the potential clinical application of this method.

The primary limitation of this study is that arthroscopy was not used to confirm the imaging-based diagnosis. In practice, very few patients required diagnostic arthroscopy, particularly in light of the widespread use of MRI in routine clinical practice. The main goal of this study was to evaluate the image quality of ACS accelerated 2D routine sequences for the diagnosis of ankle injuries. Additional research with larger sample size and arthroscopy confirmation may be necessary to confirm whether there is a difference between the clinical findings identified using the ACS *versus* PI and CS sequences. Additionally, it was not investigated whether these ACS sequences could be applied to MRI systems with a weaker magnet.

To summarize, this study presented a structured approach to reduce scan time for MRI of the ankle. We concluded that using ACS acceleration factors of 3.2–3.3× to acquire 2D FSE sequences of the ankle is feasible, with a reduction in scan time of 32–43 % comparing to CS 2.1× and PI 2.0×, without significant decrease in diagnostic performance. The ACS acceleration is a reliable alternative to conventional PI and CS and could potentially enhance the productivity of MRI systems and patient comfort in musculoskeletal radiological practices.

### Supplementary Information

**Additional file 1: Table S1.** Signal-to-noise ratio of ligament, cartilage, subchondral bone, tendon, fluid, muscle, and fat. **Table S2.** ANOVA and post-hoc analysis results for signal-to-noise ratio of the structures ligament, cartilage, subchondral bone, tendon, fluid, muscle, and fat. **Table S3.** Contrast-to-noise ratio of ligament/fluid, ligament/fat, cartilage/fluid, cartilage/subchondral bone, tendon/fluid, and tendon/muscle. **Table S4.** ANOVA and post-hoc analysis results for contrast-to-noise ration of ligament/fluid, ligament/fat, cartilage/fluid, cartilage/subchondral bone, tendon/fluid, and tendon/muscle.

## Data Availability

The data that support the findings of this study are not openly available due to reasons of sensitivity and are available from the corresponding author upon reasonable request.

## Abbreviations

2D: Two-dimensional
ACS: AI-assisted compressed sensing
AI: Artificial intelligence
CNN: Convolutional neural network
CNR: Contrast-to-noise ratio
CS: Compressed sensing
Fat-sat: Fat-saturated
FSE: Fast spin-echo
GAN: Generative adversarial network
MRI: Magnetic resonance imaging
PD: Proton density
PI: Parallel imaging
ROI: Region of interest
SD: Standard deviation
SI: Signal intensity
SNR: Signal-to-noise ratio
