# Supplementary material for: AI-assisted accelerated MRI of the ankle: clinical practice assessment
Source: Eur Radiol Exp. 2023 Oct 20;7:62. doi: 10.1186/s41747-023-00374-5 (PMC10587051; doi:10.1186/s41747-023-00374-5)
Supplement: Supplementary file 1 — Additional file 1: Table S1. Signal-to-noise ratio of ligament, cartilage, subchondral bone, tendon, fluid, muscle, and fat. Table S2. ANOVA and post-hoc analysis results for signal-to-noise ratio of the structures ligament, cartilage, subchondral bone, tendon, fluid, muscle, and fat. Table S3. Contrast-to-noise ratio of ligament/fluid, ligament/fat, cartilage/fluid, cartilage/subchondral bone, tendon/fluid, and tendon/muscle. Table S4. ANOVA and post-hoc analysis results for contrast-to-noise ration of ligament/fluid, ligament/fat, cartilage/fluid, cartilage/subchondral bone, tendon/fluid, and tendon/muscle. [file 41747_2023_374_MOESM1_ESM.docx]

**AI-assisted accelerated MRI of the ankle: clinical practice assessment**

**ELECTRONIC SUPPLEMENTARY MATERIAL**

**Table S1.** Signal-to-noise ratio of ligament, cartilage, subchondral bone, tendon, fluid, muscle, and fat

| **PD-weighted fat-sat transversal** | | | | | | | |
| --- | --- | --- | --- | --- | --- | --- | --- |
|  | Ligament | Cartilage | Subchondral bone | Tendon | Fluid | Muscle | Fat |
| ACS | 1.40±0.41 | 5.22±1.23 | 3.64±0.63 | 2.16±0.55 | 24.39±7.32 | 16.95±2.90 | 8.09±2.04 |
| CS | 1.60±0.93 | 5.28±2.77 | 3.19±0.42 | 2.49±0.66 | 21.20±5.87 | 14.01±2.11 | 6.49±1.43 |
| PI | 1.56±0.48 | 4.93±1.02 | 3.38±1.46 | 2.81±0.90 | 20.41±5.98 | 13.99±2.09 | 6.47±1.41 |
| **PD-weighted fat-sat sagittal** | | | | | | | |
|  | Cartilage | Subchondral bone | Tendon | Fluid | Muscle | Fat |  |
| ACS | 5.46±1.23 | 8.90±1.81 | 3.14±0.48 | 29.63±14.47 | 21.38±5.82 | 12.65±4.71 |  |
| CS | 5.37±1.20 | 7.72±1.31 | 4.06±0.56 | 29.43±16.10 | 20.42±5.43 | 10.82±3.63 |  |
| PI | 5.19±1.14 | 7.05±0.92 | 4.22±0.57 | 28.17±15.01 | 19.16±4.86 | 10.11±2.92 |  |
| **PD-weighted fat-sat coronal** | | | | | | | |
|  | Cartilage | Subchondral bone | Tendon | Fluid | Muscle | Fat |  |
| ACS | 6.13±1.51 | 6.96±0.77 | 3.55±0.60 | 31.62±14.68 | 22.92±5.27 | 10.38±2.24 |  |
| CS | 6.33±4.03 | 6.62±0.85 | 5.13±1.00 | 31.88±15.45 | 21.07±4.81 | 9.21±2.04 |  |
| PI | 5.87±1.44 | 6.68±0.73 | 5.41±1.18 | 30.33±14.70 | 20.15±4.27 | 9.15±1.81 |  |
| **T2-weighted transversal** | | | | | | | |
|  | Ligament | Cartilage | Subchondral bone | Tendon | Fluid | Muscle | Fat |
| ACS | 1.74±0.57 | 3.41±1.04 | 5.30±1.82 | 3.35±0.91 | 30.53±14.53 | 9.98±2.46 | 27.84±12.18 |
| CS | 1.92±0.85 | 3.56±1.12 | 5.41±1.88 | 4.72±1.71 | 29.98±15.68 | 9.32±2.05 | 26.34±10.78 |
| PI | 1.99±0.71 | 3.43±1.08 | 5.14±2.93 | 4.62±1.56 | 28.1±14.65 | 9.46±2.07 | 25.75±10.60 |
| **T1-weighted coronal** | | | | | | | |
|  | Cartilage | Subchondral bone | Tendon | Fluid | Muscle | Fat |  |
| ACS | 9.94±2.16 | 14.94±3.18 | 3.57±1.08 | 13.51±3.41 | 19.46±3.80 | 29.93±9.84 |  |
| CS | 9.98±1.86 | 14.2±3.02 | 3.88±2.66 | 11.35±2.47 | 17.54±3.41 | 29.82±9.78 |  |
| PI | 9.41±2.09 | 13.12±2.76 | 4.13±2.71 | 10.71±2.53 | 16.75±3.34 | 28.70±9.09 |  |

Data given as means ± standard deviations.  *ACS*, Artificial intelligence-assisted compressed sensing, *CS* Compressed sensing, *PD* Proton density, *PI* Parallel imaging.

**Table S2.** ANOVA and post-hoc analysis results for signal-to-noise ratio of the structures ligament, cartilage, subchondral bone, tendon, fluid, muscle, and fat.

| **PD fat-sat transversal** | | | | | | | |
| --- | --- | --- | --- | --- | --- | --- | --- |
|  | Ligament | Cartilage | Subchondral bone | Tendon | Fluid | Muscle | Fat |
| ANOVA | <0.0001 | 0.0005 | <0.0001 | <0.0001 | <0.0001 | <0.0001 | <0.0001 |
| ACS vs CS | 0.0001 | 0.0655 | <0.0001 | <0.0001 | <0.0001 | <0.0001 | <0.0001 |
| ACS vs PI | <0.0001 | 0.0003 | <0.0001 | <0.0001 | <0.0001 | <0.0001 | <0.0001 |
| CS vs PI | 0.0504 | 0.3553 | 0.0075 | <0.0001 | 0.2630 | 0.4712 | >0.9999 |
| **PD fat-sat Sag** | | | | | | | |
|  | Cartilage | Subchondral bone | Tendon | Fluid | Muscle | Fat |  |
| ANOVA | <0.0001 | <0.0001 | <0.0001 | 0.0018 | <0.0001 | <0.0001 |  |
| ACS vs CS | 0.0087 | <0.0001 | <0.0001 | >0.9999 | 0.4712 | <0.0001 |  |
| ACS vs PI | <0.0001 | <0.0001 | <0.0001 | 0.0011 | <0.0001 | <0.0001 |  |
| CS vs PI | 0.0005 | <0.0001 | 0.7616 | 0.0002 | <0.0001 | <0.0001 |  |
| **PD fat-sat coronal** | | | | | | | |
|  | Cartilage | Subchondral bone | Tendon | Fluid | Muscle | Fat |  |
| ANOVA | 0.0048 | <0.0001 | <0.0001 | 0.0018 | <0.0001 | <0.0001 |  |
| ACS vs CS | 0.0954 | <0.0001 | <0.0001 | >0.9999 | <0.0001 | <0.0001 |  |
| ACS vs PI | 0.0042 | <0.0001 | <0.0001 | 0.0045 | <0.0001 | <0.0001 |  |
| CS vs PI | 0.8824 | 0.0254 | 0.0054 | 0.0087 | <0.0001 | >0.9999 |  |
| **T2 transversal** | | | | | | | |
|  | Ligament | Cartilage | Subchondral bone | Tendon | Fluid | Muscle | Fat |
| ANOVA | <0.0001 | <0.0001 | <0.0001 | <0.0001 | <0.0001 | <0.0001 | 0.0003 |
| ACS vs CS | 0.0008 | <0.0001 | <0.0001 | <0.0001 | 0.2130 | <0.0001 | 0.0016 |
| ACS vs PI | <0.0001 | >0.9999 | <0.0001 | <0.0001 | <0.0001 | 0.0003 | 0.0015 |
| CS vs PI | 0.0271 | 0.0002 | <0.0001 | 0.5239 | 0.0032 | 0.7545 | >0.9999 |
| **T1 coronal** | | | | | | | |
|  | Cartilage | Subchondral bone | Tendon | Fluid | Muscle | Fat |  |
| ANOVA | <0.0001 | <0.0001 | <0.0001 | <0.0001 | <0.0001 | <0.0001 |  |
| ACS vs CS | >0.9999 | <0.0001 | 0.0035 | <0.0001 | <0.0001 | >0.9999 |  |
| ACS vs PI | <0.0001 | <0.0001 | <0.0001 | <0.0001 | <0.0001 | 0.0005 |  |
| CS vs PI | <0.0001 | <0.0001 | <0.0001 | 0.0069 | <0.0001 | <0.0001 |  |

*ACS*, Artificial intelligence-assisted compressed sensing, *CS* Compressed sensing, *FSE* Fast spin-echo, *PD* Proton density, *PI* Parallel imaging.

**Table S3.** Contrast-to-noise ratio of ligament/fluid, ligament/fat, cartilage/fluid, cartilage/subchondral bone, tendon/fluid, and tendon/muscle.

| **PD-weighted fat-sat transversal** | | | | | | |
| --- | --- | --- | --- | --- | --- | --- |
|  | Cartilage/Fluid | Cartilage/  Subchondral Bone | Tendon/Fluid | Tendon/  Muscle | Ligament/Fluid | Ligament/Fat |
| ACS | 4.93±1.41 | 3.53±0.78 | 19.60±4.97 | 10.56±2.38 | 13.41±3.35 | 1.91±1.00 |
| CS | 4.69±1.35 | 3.34±0.68 | 16.81±4.11 | 8.51±1.79 | 12.21±3.13 | 1.53±0.97 |
| PI | 4.54±1.23 | 3.25±0.70 | 16.13±4.09 | 8.35±1.81 | 11.86±2.71 | 1.46±0.79 |
| **PD-weighted fat-sat sagittal** | | | | | | |
|  | Cartilage/Fluid | Cartilage/ Subchondral Bone | Tendon/Fluid | Tendon/  Muscle |  |  |
| ACS | 5.91±1.89 | 3.49±0.92 | 23.47±9.73 | 12.38±3.36 |  |  |
| CS | 5.87±1.77 | 3.56±0.90 | 23.45±10.33 | 11.96±3.06 |  |  |
| PI | 5.52±1.71 | 3.40±0.87 | 21.82±9.76 | 11.14±2.98 |  |  |
| **PD-weighted fat-sat coronal** | | | | | | |
|  | Cartilage/Fluid | Cartilage/ Subchondral Bone | Tendon/Fluid | Tendon/  Muscle |  |  |
| ACS | 6.98±2.19 | 4.16±1.04 | 27.71±11.04 | 16.75±3.43 |  |  |
| CS | 7.30±3.54 | 4.40±3.66 | 27.79±11.79 | 15.62±3.11 |  |  |
| PI | 6.81±2.20 | 4.55±6.23 | 26.28±11.30 | 14.75±2.90 |  |  |
| **T2-weighted transversal** | | | | | | |
|  | Cartilage/Fluid | Cartilage/ Subchondral Bone | Tendon/Fluid | Tendon/  Muscle | Ligament/Fluid | Ligament/Fat |
| ACS | 10.19±3.36 | 3.13±1.07 | 26.35±11.25 | 6.64±1.76 | 16.51 ± 7.34 | 15.19 ± 5.53 |
| CS | 10.10±3.48 | 2.92±1.08 | 25.25±12.01 | 5.40±1.55 | 15.84 ± 7.53 | 14.01 ± 5.48 |
| PI | 9.20±3.13 | 2.66±0.97 | 23.26±10.63 | 5.39±1.56 | 14.87 ± 6.24 | 14.03 ± 5.41 |
| **T1-weighted coronal** | | | | | | |
|  | Cartilage/Fluid | Cartilage/ Subchondral Bone | Tendon/Fluid | Tendon/  Muscle |  |  |
| ACS | 1.42±0.76 | 6.82±1.41 | 7.71±1.98 | 10.29±2.50 |  |  |
| CS | 1.65±0.72 | 7.06±1.43 | 6.60±1.58 | 9.57±2.21 |  |  |
| PI | 1.53±0.68 | 6.53±1.35 | 6.23±1.53 | 9.17±2.18 |  |  |

Data given as means ± standard deviations.  *ACS*, Artificial intelligence-assisted compressed sensing, *CS* Compressed sensing, *PD* Proton density, *PI* Parallel imaging.

**Table S4.** ANOVA and post-hoc analysis results for contrast-to-noise ration of ligament/fluid, ligament/fat, cartilage/fluid, cartilage/subchondral bone, tendon/fluid, and tendon/muscle.

| **PD fat-sat transversal** | | | | | | |
| --- | --- | --- | --- | --- | --- | --- |
|  | Cartilage/  Fluid | Cartilage/  Subchondral bone | Tendon/Fluid | Tendon/  Muscle | Ligament/  Fluid | Ligament/Fat |
| ANOVA | <0.0001 | <0.0001 | <0.0001 | <0.0001 | <0.0001 | <0.0001 |
| ACS vs CS | <0.0001 | <0.0001 | <0.0001 | <0.0001 | <0.0001 | <0.0001 |
| ACS vs PI | <0.0001 | <0.0001 | <0.0001 | <0.0001 | <0.0001 | <0.0001 |
| CS vs PI | 0.0203 | 0.0063 | 0.0008 | 0.0032 | 0.0940 | 0.5880 |
| **PD fat-sat sagittal** | | | | | | |
|  | Cartilage/  Fluid | Cartilage/ Subchondral Bone | Tendon/Fluid | Tendon/  Muscle |  |  |
| ANOVA | <0.0001 | <0.0001 | <0.0001 | <0.0001 |  |  |
| ACS vs CS | >0.9999 | <0.0001 | >0.9999 | 0.0744 |  |  |
| ACS vs PI | <0.0001 | >0.9999 | <0.0001 | <0.0001 |  |  |
| CS vs PI | <0.0001 | <0.0001 | <0.0001 | <0.0001 |  |  |
| **PD fat-sat coronal** | | | | | | |
|  | Cartilage/  Fluid | Cartilage/ Subchondral Bone | Tendon/Fluid | Tendon/  Muscle |  |  |
| ANOVA | 0.0005 | 0.0097 | 0.0002 | <0.0001 |  |  |
| ACS vs CS | 0.9515 | 0.0792 | >0.9999 | <0.0001 |  |  |
| ACS vs PI | 0.0175 | 0.0111 | 0.0012 | <0.0001 |  |  |
| CS vs PI | 0.0005 | >0.9999 | 0.0011 | <0.0001 |  |  |
| **T2 trasversal** | | | | | | |
|  | Cartilage/  Fluid | Cartilage/ Subchondral Bone | Tendon/Fluid | Tendon/  Muscle | Ligament/  Fluid | Ligament/Fat |
| ANOVA | <0.0001 | <0.0001 | <0.0001 | <0.0001 | 0.0015 | <0.0001 |
| ACS vs CS | >0.9999 | <0.0001 | 0.0006 | <0.0001 | 0.0472 | <0.0001 |
| ACS vs PI | <0.0001 | <0.0001 | <0.0001 | <0.0001 | 0.0013 | <0.0001 |
| CS vs PI | <0.0001 | <0.0001 | 0.0004 | >0.9999 | 0.8168 | >0.9999 |
| **T1 coronal** | | | | | | |
|  | Cartilage/  Fluid | Cartilage/ Subchondral Bone | Tendon/Fluid | Tendon/  Muscle |  |  |
| ANOVA | <0.0001 | <0.0001 | <0.0001 | <0.0001 |  |  |
| ACS vs CS | <0.0001 | <0.0001 | <0.0001 | <0.0001 |  |  |
| ACS vs PI | 0.0021 | <0.0001 | <0.0001 | <0.0001 |  |  |
| CS vs PI | <0.0001 | <0.0001 | <0.0001 | <0.0001 |  |  |

*ACS*, Artificial intelligence-assisted compressed sensing, *CS* Compressed sensing, *FSE* Fast spin-echo, *PD* Proton density, PI Parallel imaging.
